# Dendrimers and Dendritic Materials against Infectious Diseases

**DOI:** 10.3390/pharmaceutics14010154

**Published:** 2022-01-10

**Authors:** Francisco Javier de La Mata, Paula Ortega, Sandra García-Gallego

**Affiliations:** 1 Department of Organic and Inorganic Chemistry, Faculty of Sciences, and Research Institute in Chemistry “Andrés M. del Río” (IQAR), University of Alcalá, 28801 Alcalá de Henares, Spain; paula.ortega@uah.es (P.O.); sandra.garciagallego@uah.es (S.G.-G.); 2 Institute Ramón y Cajal for Health Research (IRYCIS), 28034 Madrid, Spain

The COVID-19 pandemic showed more deeply the need of our society to provide new therapeutic strategies to fight infectious diseases, not only against currently known illnesses, where common antibiotics and drugs appear to be not fully effective, but also against new infectious threats that may arise. 

In a context where antibiotic resistance and the spread of new viruses are seriously threatening human health, the use of nanotechnology and, in particular, dendrimers and dendritic materials is emerging as a promising strategy. Their versatility, multivalency, and on-demand design enable an efficient response to combat multiple infectious diseases. This Special Issue serves to highlight and capture the current progress in the design and use of dendritic materials in the fight against infectious diseases. It comprises a series of 8 research articles and 4 review articles, which provide a broad overview of cutting-edge strategies to beat virus, bacteria, fungi, and parasites. 

Heredero–Bermejo et al. [1] evaluated the antiamoebic properties of different cationic carbosilane dendrimers against *Acanthamoeba polyphaga*, a causative agent of the severe ocular disease keratitis. The study performed by scanning and transmission electron microscopy confirmed the dramatic alterations on both the cellular ultrastructure and the plasma membrane, as well as the subsequent impact on the trophozoite and cyst death. The most promising candidate, dendrimer [G_1_O_3_(S-NH_3_)_6_]^6+^, exhibited a significant activity with IC_50_ of 2.4 + 0.1 mg/L. Overall, this work confirmed the role of carbosilane dendrimers as a promising therapy against keratitis at concentrations that are well tolerated by the human host cells. 

Sanz del Olmo et al. [2] designed a novel family of polyphenolic carbosilane dendrimers, decorated with ferulic, caffeic, and gallic acids. The results obtained from spectrophotometric and electrochemical techniques confirmed that dendritic polyphenols exhibited higher antioxidant activities than free polyphenols. The dendrimer generation as well as the nature of the acid significantly affected the antioxidant activity and the antibacterial properties. In particular, G_1_-(Gallic)_4_ appeared as the most promising candidate, with MIC_50_ of 4 ppm against *S. aureus* and 16 ppm against *E. coli*. The potential of polyphenolic dendrimers in cosmetics field was clearly stated. 

Zhang et al. [3] reported a new approach towards metal-free paramagnetic contrast agents for Magnetic Resonance Imaging, based on highly water-soluble olygoethylene glycol dendrimers functionalized with PROXYL radicals. The first-generation dendrimer, decorated with 20 PROXYL units, exhibited a *r*_1_ relaxivity value (3.4 mM^−1^s^−1^) quite similar to that of gadolinium-diethylenetriamine pentaacetic acid (Gd-DTPA) used in clinics, but with no cytotoxicity risks due to Gd accumulation in the body. The study revealed the potential of radical dendrimers for the diagnosis and follow-up of infectious diseases. 

Vossen et al. [4] decorated PEGylated dendritic polyglycerols with mannose units and explored their ability as nanocarriers targeting macrophages as hosts of *Leishmania* parasites. The study demonstrated that these nanocarriers were endocytosed through different pathways, colocalized with the parasites at the phagolysosomes and selectively delivered the drug Amphotericin B at acidic pH. In conclusion, these nanocarriers represent a promising specific drug-delivery vehicle against leishmaniasis, as they are preferably taken up by parasite-infected macrophages. 

Heredero–Bermejo et al. [5] screened a library of cationic carbosilane dendrimers against *Candida albicans* biofilms, currently resistant against most antifungal agents. A candidate, BDSQ024, exhibited promising activity against both the formation of the biofilm as well as the elimination of existing biofilm at 16 mg/L, severely damaging fungal cells. Importantly, synergy with common antifungal drugs was found: drug concentration was clearly reduced for amphotericin (0.06 mg/L) and caspofungin (0.007 mg/L), using dendrimer BDSQ024 at nontoxic concentration (4 mg/mL). This work suggests that combination therapy using dendrimers and antifungal drugs may be an effective approach against candidiasis, reducing biofilm formation and drug resistance. 

Rodríguez-Prieto et al. [6] demonstrated the antibacterial activity of different carbosilane dendritic molecules containing silver(I) *N*-heterocyclic carbenes as well as their precursors. The impact of parameters such as topology, generation, hydrophobicity, and presence of silver ions, was explored and confirmed the crucial role of the lipophilic-hydrophilic balance. Additionally, the in-depth study to unravel the mode of action in *Bactillus subtilis* identified the cell envelope as the main target. Overall, this work highlights the potential of cationic imadazolium dendrimers or Ag(I)-NHC metallodendrimers in the fight against bacterial infections.

San Anselmo et al. [7] designed four different amphiphilic Janus dendrimers comprising 2,2′-bis(hydroxymethyl)propionic acid (bis-MPA) and 2,2′-bis(glyciloxy)propionic acid (bis-GMPA) scaffolds and hydrophilic or lipophilic moieties at their periphery. These dendrimers formed spherical or cylindrical micelles in water, with a critical aggregation concentration in the 10^−5^ M order of magnitude. The nanocarriers increased the water solubility and antiviral activity of encapsulated iopanoic acid and tiratricol, two bioactive allosteric inhibitors of Hepatitis C viral NS3 protease, confirming the potential of dendritic nanocarriers for the treatment of viral infections.

Fan et al. [8] designed antibacterial hydrogels based on the self-assembly of cationic polyester bis-MPA dendrimers and carboxylated cellulose nanofibrils (CNFs). Bis-MPA dendrimers were synthesized through the highly efficient fluoride promoted esterification (FPE) chemistry and subsequently reacted with CNFs. Hydrogels from G3 and G4 dendrimers showed 100% killing efficiency towards Gram-positive and Gram-negative bacteria. Additionally, they exhibited a high biocompatibility towards cells, arising from the role of CNFs that slowed down the release of toxic cationic dendrimers. Hybrid dendritic hydrogels thus appear as promising antibacterial materials for the treatment of bacterial infections.

Excellent reviews are also included in this Special Issue, highlighting the importance of dendrimers and dendritic materials as new tools for combating infectious diseases.

Ortega et al. [9] provide the reader with a general overview about the uses of dendrimers and dendritic materials in the treatment, prevention, and diagnosis of highly prevalent infectious diseases and their advantages compared to that of traditional approaches. Examples of dendrimers as antimicrobial agents *per se*, as nanocarriers of antimicrobial drugs, as well as their uses in gene transfection, in vaccines or as contrast agents in imaging assays, are presented. 

Falanga et al. [10] focus on the impact of dendritic nanoplatforms based on antimicrobial, antiviral and cell penetrating peptides, as potent broad-spectrum agents. The review provides an interesting overview of the main concepts involved in the design of antimicrobial peptides for dendrimer decoration, aiming to promote the clinical translation of these promising conjugates.

Folliero et al. [11] review the potential of dendrimers for therapeutic, prophylactic, and diagnostic purposes towards parasitic infections, including malaria, leishmaniasis, schistosomiasis, toxoplasmosis, and acanthamebiasis. Despite still being in its infancy, this field holds great promise and future developments are foreseeable.

Finally, Mignani et al. [12] offer an interesting overview about the use of biocompatible dendrimers to combat SARS-CoV-2 virus infections. Strategies as nanocarriers and as drugs per se are presented, as well as diagnostic tools. While there are still few clinical examples, dendrimers present limitless possibilities to decrease the burden of COVID-19 disease. 

All these articles will help researchers to understand the state of the art in this scientific field and will undoubtedly support the development of innovative approaches to fight against microbial diseases. We hope that this special issue in Pharmaceutics will be of interest to a wide number of readers. 
